# Osteonecrosis of the Femoral Head in an 8-Year Old Boy: Appraisal of Evolution by DXA

**DOI:** 10.5334/jbr-btr.990

**Published:** 2015-12-30

**Authors:** Stavroula J. Theodorou, Daphne J. Theodorou, Yousuke Kakitsubata

**Affiliations:** 1 Department of Radiology, University Hospital of Ioannina, Ioannina, Greece; 2 Department of Radiology, General Hospital of Ioannina, Ioannina, Greece and National Healthcare System, Greece; 3 Department of Radiology, Miyazaki Konan Hospital, Miyazaki, Japan

**Keywords:** Osteonecrosis, Dual energy X-ray absorptiometry, Osteoporosis, Bone Mineral Density

## Abstract

Assessment of skeletal status and bone mineral density (BMD) in the pediatric population requires knowledge of the child’s overall health, clinical history of chronic illness and/or risk factors for osteoporosis, and atraumatic fractures. Dual-energy X-ray absorptiometry (DXA) is the gold standard for the assessment of bone health, in children and adolescents. The interpreting physician needs to acknowledge that diagnosis of low BMD in growing subjects should include in addition to densitometric measurements, the synchronous assessment of the DXA-generated image for collateral findings that may cause erroneous evaluation of bone mass and improper management.

## Introduction

Osteonecrosis refers to the in situ death of bone [1] and usually affects the femoral head [2,3]. Epiphyseal osteonecrosis has been documented in patients treated with steroid medication for various underlying diseases, and in leukemic patients prior to such therapy. Osteonecrosis in patients with leukemia most commonly involves the femoral epiphyses and condyles and the proximal portion of the humerus. Radiation therapy of the total body, with or without chemotherapy, may be employed prior to transplantation in the treatment of childhood leukemia. Osteonecrosis is one of the many bone sequelae of total body irradiation, which may be more severe in children in the first decade of life [1]. Progressive changes of osteonecrosis in the hips may result in false elevation of bone mineral density (BMD).

Herein we report the case of a young child with osteonecrosis of both hips who underwent dual energy X-ray absorptiometry (DXA) to evaluate bone mass. Although it allowed evaluation of evolution of osteonecrosis over time, this case clearly indicates that osteonecrosis can be another source of diagnostic error causing artifactual elevation of the BMD measurements. The teaching point of this pediatric case is that visual inspection of DXA images should always precede interpretation of the densitometric data.

## Case report

An 8-year-old boy with a history of 5 years of acute lymphoblastic leukemia was referred for radiographic evaluation of the pelvis because of left hip pain and a limp. The patient had undergone radiation therapy of the total body and bone marrow transplantation at age 6. Since the onset of the disease he had been receiving intravenous and oral corticosteroids. Three months post-transplantation, the patient developed lung disease secondary to graft- versus-host disease and received high doses of intravenous and oral steroids. Subsequently, the patient developed autoimmune hemolytic anemia and was treated again with high doses of intravenous and oral steroids.

**Figure 1:**
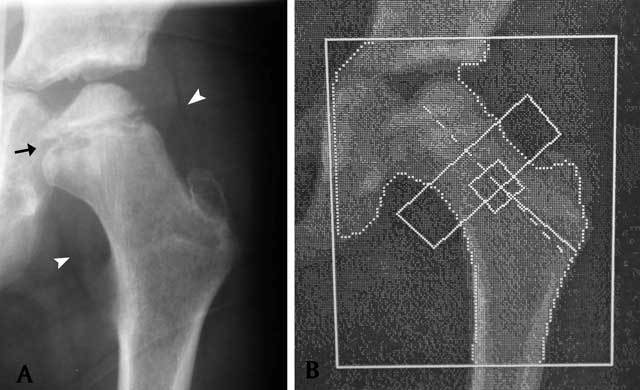
(A) The initial frontal radiograph of the left hip at 8-year age (phase of disease onset) shows early abnormalities including a sclerotic femoral ossification center that is laterally displaced, metaphyseal irregularity (arrow) with cyst formation, and mild soft tissue distortion (arrowheads). (B) DXA scan shows subtle osteosclerosis of the left hip. The BMD value of the total hip is 0.481 g/cm^2^.

**Figure 2:**
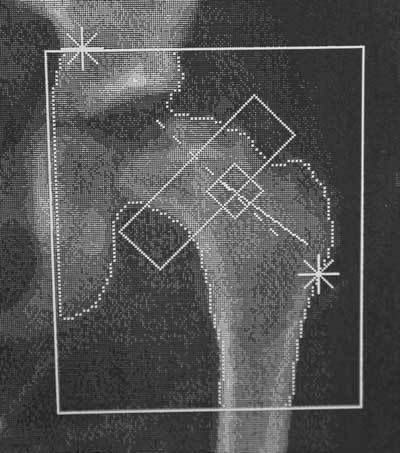
DXA scan at 9-year age (phase of fragmentation) shows further progression of osteonecrosis with collapse and loss of normal spherical configuration of the femoral head. Bone sclerosis caused false elevation of the BMD, which is now 0.567 g/cm^2^.

**Figure 3:**
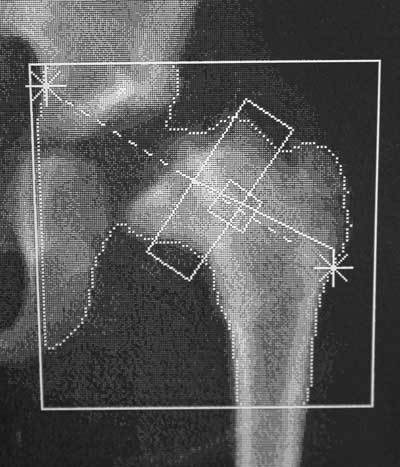
DXA scan at 10-year age (advanced osteonecrosis) shows destruction of femoral epiphysis with broad and short femoral neck. Growth of the hip has been arrested. The BMD value of the total hip is 0.788 g/cm^2^.

Physical examination revealed a small for his age Cushingoid boy with severe loss of muscle bulk due to steroid-induced myopathy. His height was 47 inches, which lies between the 3^rd^ and 9^th^ centile and his weight was 54.3 lb, which lies between the 24^th^ and 50^th^ centile. Radiographic evaluation of the spine and hips revealed compression fractures in thoracic spine (T11, T12) and mild deformity of the femoral epiphyses (Fig. 1A). Because of the history of long-term corticosteroid use, BMD was measured in the lumbar spine (L1-4) [0.485 g/cm^2^, Z-score -1.97] and proximal femur [0.481 g/cm^2^] using DXA (Fig. 1B). Trabecular BMD was measured at the 4% distal radius using peripheral quantitative computed tomography (pQCT) [109.5 mg/cm^3^, Z-score -2.2]. On the basis of established pediatric densitometric criteria [5], a diagnosis of low bone mineral content was made and patient was started on treatment with vitamin D and calcium. Follow-up DXA studies to determine response to treatment at 12 months demonstrated collapse of the femoral head and increasing BMD (Fig. 2). The lumbar spine and hip bone densities were 0.542 g/cm^2^, Z-score -1.46 and 0.567 g/cm^2^, respectively. Sequential follow-up DXA scans at 24 months showed further destruction of the femoral head and an increase in bone mass (Fig. 3). The lumbar spine and hip bone densities were 0.591 g/cm^2^, Z-score -1.35 and 0.788 g/cm^2^, respectively.

Trabecular BMD at the 4% distal radial site by pQCT at 12 and 24 months was 135.8 mg/cm^3^, Z-score -1.6 and 133.7 mg/cm^3^, Z-score -1.9, respectively. The concurrent visual assessment of the DXA images showed progressive deterioration of the left hip joint, beginning as vague osteosclerosis and becoming severe with destruction of the femoral head, suggesting the diagnosis of osteonecrosis.

## Discussion

Although DXA is a valuable tool for comprehensive skeletal assessment of children, errors in interpreting DXA results may generate significant parental concern and can result in costly and/or unnecessary use of medications, or restrictions on physical activity.

Our experience with this case highlights the importance of interpretation of DXA data by skilled professionals at pediatric densitometry centers.

Osteonecrosis of the femoral head has been documented in patients with leukemia treated with steroid medication, or even prior to such therapy [1]. Furthermore, the association of ischemic necrosis of bone and the treatment of leukemia with combination chemotherapy regimens has been reported on numerous occasions. Indeed, the role of cytotoxic drugs, in the production of ischemic necrosis of bone is unclear. The addition of radiation therapy to the total body (including the pelvis), commonly used in patients who subsequently develop osteonecrosis, suggests that the inclusion of the femoral head in the irradiation field is a significant factor in the development of ischemic necrosis [1]. The precise cause of ischemic necrosis in persons with leukemia is not known. The almost constant association with corticosteroid administration suggests that these agents are important etiologic factors. The etiology of osteonecrosis in our patient most probably was multifactorial with chronic ill-health, leukemia itself, corticosteroids, chemotherapy, and radiotherapy being implicated.

A change in bone density is the predominant feature of osteonecrosis on radiographs, where characteristic findings include arc-like subchondral radiolucent lesions, patchy lucent areas and osteosclerosis, and finally articular collapse. Indeed, the radiographic findings of osteonecrosis reflect the stage of disease, depending on the amount of bone revascularization, reparative reossification and resorption of necrotic bone [2,3]. Revascularization may result in trabecular bone resorption with radiolucent areas developing around the necrotic lesion. New bone then is deposited on the necrotic bone surface. Over months or years dead bone may be slowly resorbed. Osteosclerosis seen in osteonecrosis may represent compression of necrotic trabeculae, reactive eburnation secondary to the healing process, or lack of participation of the bone in hyperemia and osteoporosis of adjacent viable bone. As reossification of the femoral head proceeds, it will remold its shape according to impacting mechanical forces. In our patient, we observed the sequential changes of osteonecrosis of the femoral head, representing different phases of the disease by DXA. We recorded evolution of the disease which progressed over the subsequent 12 to 24 months, with significant collapse of the femoral head.

Osteoporosis associated with iatrogenic hypercortisolism, as in this case, is well known. In steroid-induced osteoporosis, histologic studies have revealed decreased bone formation due to direct corticosteroid inhibition of osteoblast formation, and increased bone resorption. This latter effect may relate to either direct stimulation of osteoclastic activity or increased parathyroid hormone secretion [4].

The clinical goals of bone densitometry are to identify patients with primary bone disease (i.e., idiopathic juvenile osteoporosis, osteogenesis imperfecta), or at risk for a secondary bone disease (i.e., inflammatory bowel disease, cystic fibrosis, cerebral palsy, endocrine disorders, hematologic disorders, inadequate nutrition, medical therapies, childhood cancer) and an elevated risk of fracture [5]. Skeletal health in youth can be assessed by DXA, quantitative computed tomography, peripheral quantitative computed tomography, quantitative ultrasonography, MR imaging, or radiogrammetry. DXA is now established as the state-of-the-art modality for noninvasive assessment of bone density because of its availability, reproducibility, speed, and low exposure to ionizing radiation [6,7]. Because of the relatively high resolution of fan-beam DXA scanners, anatomical details of the examined region may be clearly depicted on the DXA generated images [6,7,8]. Correlation with radiography may be required however, to better define the disease process and to exclude potential sources of diagnostic error. This is owing to the fact that both resolution and signal-to-noise ratio of DXA scans are inferior to those of radiographs, and as such DXA can only supplement and not rather replace radiographs [6,7,8]. As a rule of thumb, the scan images on the DXA printout need to be carefully reviewed for the presence of potential sources of diagnostic error such as improper patient positioning and placement of regions of interest (ROIs), osteosclerosis, metallic items, fractures and degenerative disease [7,8,9]. Additional sources of error include motion artifacts, retained intestinal barium, nasogastric tube, spinal scoliosis, and spinal orthopedic hardware.

In this complex pediatric clinical case, BMD as measured by DXA was found to spuriously increase on the follow-up scans. Osteonecrosis of the femoral head was the cause of false elevation of the BMD, in our patient. If this pitfall was not recognized, this patient with low BMD would be misdiagnosed as responding to treatment over time. Our case highlights the importance of analysis of DXA images in addition to numeric data for identification of the variable sources of diagnostic error, including osteonecrosis of the femoral head.

## Competing Interests

The authors declare that they have no competing interests.
